# Time trend analysis of Injury Severity score of adult trauma patients with emergent CT examination

**DOI:** 10.1007/s10140-024-02253-x

**Published:** 2024-06-17

**Authors:** Stefanie Neef, Felix G. Meinel, Roberto Lorbeer, Felix Ammermann, Marc-André Weber, Manuela Brunk, Philipp Herlyn, Ebba Beller

**Affiliations:** 1 Department of Anesthesiology, Intensive Care Medicine and Pain Management, Helios Weißeritztal- Kliniken, Klinikum Freital, Germany; 2 Institute of Diagnostic and Interventional Radiology, Pediatric Radiology and Neuroradiology, University Medical Centre Rostock, Ernst-Heydemann-Str. 6, 18057 Rostock, Germany; 3 Department of Radiology, University Hospital, LMU Munich, Munich, Germany; 4 Department of Trauma, Hand and Reconstructive Surgery, University Medical Center Rostock, Rostock, Germany; 5 Clinic for Trauma, Reconstructive and Hand Surgery, Municipal Clinic Dresden, Dresden, Germany; 6 Department of Pedatrics, University Children’s Hospital, Klinikum Oldenburg AäR, Rahel-Srauß-Street 10., 26133 Oldenburg, Germany

**Keywords:** Computed tomography, Injury severity score, Trauma, Whole-body CT

## Abstract

**Purpose:**

Controversy exists about whole-body computed tomography (CT) as a primary screening modality for suspected multiple trauma patients. Therefore, the aim of this study was to analyze time trends of CT examinations for trauma patients in relation to the Injury Severity Score (ISS).

**Methods:**

We retrospectively analyzed 561 adult trauma patients (mean age = 54 years) who were admitted to the trauma room of our hospital, immediately followed by a CT examination, in 2009, 2013 und 2017. Review of electronic patient charts was performed to determine the cause of injury. ISS was either calculated upon hospital charts and CT imaging reports or documented in the TraumaRegister DGU® for trauma patients with ICU treatment or ISS ≥ 16.

**Results:**

An increasing number of CT examinations of acute trauma patients were performed at our hospital with 117 patients in 2009 compared to 192 in 2013 and 252 in 2017. Their mean age increased (50 years in 2009, 54 in 2013 and 55 in 2017;*p* = 0.046), whereas their mean ISS decreased over time (15.2 in 2009 compared to 12.1 in 2013 and 10.6 in 2017;*p* = 0.001), especially in women (15.1 in 2009, 11.8 in 2013 and 7.4 in 2017;*p* = 0.001 both), younger age groups (18 to 24 years:15.6 in 2009, 6.5 in 2013 and 8.9 in 2017; *p* = 0.033 and 25 to 49 years:15.0 in 2009, 11.2 in 2013 and 8.3 in 2017;*p* = 0.001) as well as motor vehicle collision (MVC) victims (16.2 in 2009, 11.8 in 2013 and 6.1 in 2017; *p* < 0.001). Trauma patients with a high ISS were especially more likely of older age (OR 1.02,*p* < 0.001) and with the type of incident being a fall (< 3 m: OR3.84,*p* < 0.001;>3 m: OR6.22,*p* < 0.001) compared to MVC.

**Conclusion:**

Previous studies suggesting a benefit of primary whole-body CT for trauma patients might not reflect the current patient population with decreasing ISS. Especially females, younger age groups and MVC patients might benefit from stricter selection criteria for receiving whole-body CT. Our results also emphasize the importance of prevention of fall or tumble for elderly people.

## Background

Computed tomography (CT) imaging is an important source of information to guide clinical management in trauma patients and has increased drastically in emergency departments over the years [1–3]. CT trauma protocol varies by institution with some conducting a contrast-enhanced whole-body CT (WBCT) scan for every trauma patient, while others may decide between WBCT and a CT scan of selective body region(s) [4, 5]. However, despite supporting improved accuracy of diagnosis by CT imaging, the increase in emergency radiology volume has also been criticized as replacing physical examination, increasing radiation exposure, and enabling defensive medicine practices [6].

Although CT utilization has increased in emergency departments, no equal increase in the prevalence of life-threatening conditions was found between 1998 and 2007 [7]. Controversy especially exists about WBCT as a primary screening modality for suspected multiple trauma patients. Results of a large-scale registry (TraumaRegister DGU®) of the German Trauma society in 2009 and 2013 suggest a significantly decreased risk-adjusted ratio of observed to expected deaths with primary WBCT [8, 9]. However, in 2016, the only prospective randomized trial (REACT-2) about the benefit of WBCT so far reported no reduction in mortality comparing immediate WBCT to conventional imaging plus selective CT for trauma patients suspected of having severe or life-threatening injuries [10].

Thus, the aim of this study was to evaluate the use of CT examinations for trauma patients in 2009, 2013 and 2017 in relation to the Injury Severity Score (ISS). This might be important to optimize utilization of CT imaging in these patients, which includes the reduction of unnecessary radiation exposure and minimizing unnecessary strain on healthcare resources.

## Methods

### Patient selection and study design

This study was designed as a retrospective, single-center trend study. Patients were identified by retrospective search of our radiology information system (Centricity 5.0, GE Healthcare, Barrington, Illinois). Inclusion criteria were adult trauma patients (age ≥ 18 years) and CT examination during trauma room treatment at our hospital over a 1-year period, including the year 2009, 2013 und 2017. Trauma CT examinations during trauma room treatment were usually performed about 30 min after hospital admission. We excluded patients younger than 18 years, with no traumatic injury (e.g. stroke or myocardial infarction) and missing clinical information. Review of electronic patient charts was performed to determine the cause of injury. The study protocol was approved by the responsible institutional review board (blinded) with waiver of informed consent. The study was conducted in compliance with the Declaration of Helsinki in its current form.

### CT technique and image analysis

All trauma CT examinations were performed on a 64-slice CT scanner (Aquilion 64, Toshiba Medical Systems, New York), which was located in a room adjacent to the trauma bay. Per institutional standard, a WBCT protocol for trauma patients included a non-enhanced CT of the head and neck with arms alongside the trunk, followed by a contrast-enhanced CT (*Imeron*® *400* mg/mL, Bracco Imaging, Milan, Italy) of the chest (arterial phase) as well as abdomen and pelvis to lesser trochanters (porto-venous phase) immediately after raising the arms alongside the head. Patients were classified as having selective CT when they received scans of one or more body regions, including head, neck, chest, abdomen/pelvis and extremities but not a whole-body CT. Both types of imaging were routinely used in trauma patient management and the decision was usually made by the trauma leader. CT datasets of all 561 patients were archived in our PACS software (IMPAX 6.5.3, Agfa HealthCare, Bonn, Germany) and re-evaluated by two readers in consensus (one medical student, one board-certified radiologist, initials blinded) to determine the type of received CT-scan (whole-body vs. selective CT).

### Abbreviated injury scale (AIS) and injury severity score (ISS)

AIS and ISS values of trauma patients with intensive care unit (ICU) treatment or an ISS ≥ 16 were documented in the TraumaRegister DGU®, which is a prospective, multicenter, standardized and anonymized database. As a participating hospital, this data was continuously (including the year 2009, 2013 and 2017) entered into a web-based data server that is hosted by the German Trauma Society and its Academy for Trauma Surgery [11]. For patients with no ICU treatment and ISS < 16, AIS and ISS were calculated upon hospital charts and CT imaging reports: In order to calculate the ISS, the body was divided into six regions: (1) head/neck; (2) face; (3) chest; (4) abdominal or pelvic contents; (5) extremities of pelvic girdle; (6) external. An Abbreviated Injury Score (AIS) was allotted to each of these body regions, according to the Abbreviated Injury Scale© 2005 Update 2008 [12]: (1) minor, (2) moderate, (3) serious, (4) severe, (5) critical, (6) lethal injury. The ISS was then calculated by taking the sum of the square root of the three most severe injuries (with the highest AIS score) with possible values ranging from 1 to 75. Patients with an AIS of 6 were automatically assigned an ISS value of 75.

### Statistical analysis

Patients’ characteristics were summarized by mean and standard deviation for continuous variables (e.g. ISS) or number and percentage for categorical variables. Differences of ISS between examination years were investigated by nonparametric test for trend across ordered groups in the whole sample and several subgroups of co-variables. Univariate comparisons between low and high ISS groups were evaluated by chi^2^-test or t-test. Multivariable adjusted factors affecting high ISS were identified by logistic regression models providing 95% confidence interval (CI). In addition, time trends of trauma characteristics were analyzed in a subgroup of all trauma patients examined by WBCT. A *p*-value of < 0.05 was considered statistically significant. Statistical analyses were performed using Stata 16.1 (Stata Corporation, College Station, TX, USA).

## Results

### Increase of trauma CT examinations and decrease of ISS over time

An increasing number of CT examinations of acute trauma patients were performed at our hospital with 117 patients in 2009 compared to 192 patients in 2013 and 252 patients in 2017 (Table 1), respectively an increase of 64% in 2013 and of 115% in 2017, when compared to 2009. In comparison, the total number of ED visits was 34.671 in 2013 and 35.463 in 2017, respectively an increase of only 2% within four years. There was no significant difference regarding which type of CT examination (the majority were whole-body CT compared to selective CT with 73% in 2009 and 63% in both 2013 and 2017; *p* = 0.121) and gender (the majority was male with 64% in 2009, 61% in 2013 and 65% in 2017; *p* = 0.706). However, the mean age of trauma patients increased significantly with time: 50 years in 2009 compared to 54 years in 2013 and 55 years in 2017 (*p* = 0.046). We also found a substantial decrease in ISS of acute trauma patients receiving CT examinations with a mean of 15.2 in 2009 compared to 12.1 in 2013 and 10.6 in 2017 (*p* = 0.001). Please find a summary of the patient characteristics in Table 1.

**Table 1:** Characteristics of patients with trauma CT examinations from 2009, 2013 and 2017

	All	2009	2013	2017	*P*-value
No. of trauma CT	561	117	192	252	
No. of ED visits	n.a.	n.a.	34.671	35.463	
of which WBCT	364 (65%)	85 (73%)	120 (63%)	159 (63%)	0.121
of which SCT	197 (35%)	32 (27%)	72 (38%)	93 (37%)	
Age*	54(± 21)	50 (± 21)	54 (± 21)	55 (± 21)	**0.046**
Gender					0.706
female	205 (37%)	42 (36%)	75 (39%)	88 (35%)	
male	356 (64%)	75 (64%)	117 (61%)	164 (65%)	
ISS*	12.1 (± 11.8)	15.2 (± 14.2)	12.1 (± 10.2)	10.6 (± 11.5)	**0.001**

*mean and standard deviation

*P*-values are from test for trend. *P*-Values < 0.05 appear bold

ED: Emergency Department

n.a.: not available

WBCT: Whole-body computed tomography

SCT: Selective computed tomography

ISS: Injury severity score

### Detailed analysis of decrease of ISS over time

**Fig. 1:**
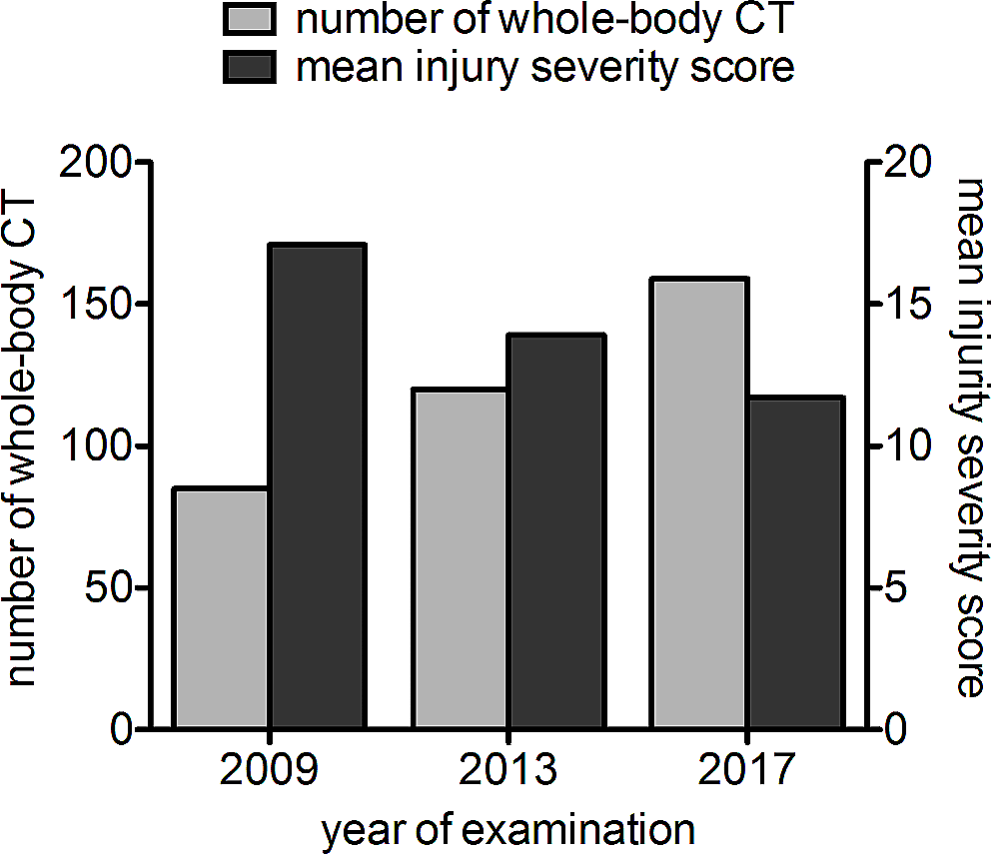
Comparison of number of WBCT and mean ISS. There was an increasing number of whole-body CT examinations of acute trauma patients performed at our hospital with 85 patients in 2009 compared to 120 patients in 2013 and 159 patients in 2017. In contrast, there was a significant decrease of the injury severity score over time in trauma patients receiving whole-body CT with a mean of 17.1 in 2009 compared to 13.9 in 2013 and 11.7 in 2017 (*p* < 0.001)

In patients receiving trauma CT examinations, a significant decrease of ISS was especially found in women (with a mean of 15.1 in 2009 compared to 11.8 in 2013 and 7.4 in 2017;*p* = 0.001 both) as well as in the younger age groups (18 to 24 years with a mean of 15.6 in 2009 compared to 6.5 in 2013 and 8.9 in 2017; *p* = 0.033 and 25 to 49 years with a mean of 15.0 in 2009 compared to 11.2 in 2013 and 8.3 in 2017; *p* = 0.001). There was a significant decrease of ISS over time in trauma patients receiving whole body CT (with a mean of 17.1 in 2009 compared to 13.9 in 2013 and 11.7 in 2017; *p* < 0.001, see also Fig. 1), but not in trauma patients receiving selective CT (*p* = 0.528). Regarding the type of incident, we only found a significant decrease in ISS in trauma patients, who suffered from a motor vehicle collision (MVC) and therefore underwent CT examination (with a mean of 16.2 in 2009 compared to 11.8 in 2013 and 6.1 in 2017; *p* < 0.001) as well as summarized under the term “others” (with a mean of 12.9 in 2013 and 32.0in 2017; *p* < 0.024). Please find more detailed information about the changes of ISS values over time in Table 2.

**Table 2:** Time Trends (from 2009, 2013 and 2017) of Mean and SD of ISS

	ISS^*^ of 2009	ISS^*^ of 2013	ISS^*^ of 2017	*P*-value
Gender				
Women	15.1 (± 14.9)	11.8 (± 11)	7.4 (± 9.0)	**0.001**
Men	15.2 (± 13.9)	12.3 (± 9.6)	12.2 (± 12.3)	0.094
Age (years)				
18–24	15.6 (± 12.8)	6.5 (± 9.9)	8.9 (± 13.3)	**0.033**
25–49	15.0 (± 13.4)	11.2 (± 8.9)	8.3 (± 9.5)	**0.001**
50–69	15.5 (± 17.3)	12.0 (± 8.5)	13.0 (± 13.1)	0.857
≥70	14.9 (± 13.4)	15.1 (± 12.3)	10.7 (± 10.4)	0.073
CT examination				
Whole-body CT	17.1 (± 15)	13.9 (± 10.3)	11.7 (± 12.3)	**< 0.001**
Selective CT	10.3 (± 10.9)	9.2 (± 9.3)	8.6 (± 9.6)	0.528
Type of incident				
Fall < 3 m	10 (± 12.6)	11.5 (± 9.8)	10.6 (± 11.4)	0.951
Fall > 3 m	22.4 (± 15.8)	13.4 (± 8.5)	15.3 (± 11.9)	0.231
Motor vehicle collision	16.2 (± 13.6)	11.8 (± 10.8)	6.1 (± 8.4)	**< 0.001**
Motorbike accident	14 (± 9.5)	10.2 (± 7.2)	16.4 (± 15)	0.955
Bicycle accident	21.1 (± 17.8)	11.8 (± 12.2)	10.8 (± 8.8)	0.293
Pedestrian accident	19.3 (± 20.9)	12.8 (± 13.1)	15.3 (± 19.8)	0.607
Other traffic accidents	-	9 (± 0)	9 (± 9.6)	0.696
Physical violence	5 (± 3.6)	8.3 (± 6.3)	6 (± 5)	0.766
Stab injury	13 (-)	4.8 (± 6.9)	7.3 (± 10.1)	0.919
Jumping	16.5 (± 17.7)	34 (-)	1 (-)	0.484
Others	-	12.9 (± 10.7)	32.0 (± 12.2)	**0.024**
Unknown	11.4 (± 12.5)	18.4 (± 10.7)	15.1 (± 12.2)	0.762

^*^mean and standard deviation

*P*-values are from test for trend. *P*-Values < 0.05 appear bold

ISS: Injury severity score

CT: computed tomography

### Predicting factors of high ISS scores

A multivariable adjusted logistic regression model was performed to identify factors associated with a high ISS (≥ 9) in trauma patients (Table 3). The risk of having an ISS ≥ 9 when receiving a trauma CT at our hospital was significantly smaller in 2017 compared to 2009 (OR 0.48, *p* = 0.009), Trauma patients with a high ISS (≥ 9) were associated with older age (OR 1.02, *p* < 0.001). When comparing to MVC, trauma patients were especially more severely injured with the type of incident being a fall < 3 m (OR 3.84, *p* < 0.001), a fall > 3 m (OR 6.22, *p* < 0.001) or unknown at the time of the CT examination (OR 9.74, *p* < 0.001). A less but still significant association was observed between high ISS and the type of incident including motorbike (OR 2.56, *p* = 0.038), bicycle (OR 3.65, *p* = 0.002), pedestrian (OR 7.46, *p* = 0.028) or other traffic accidents (OR 3.14, *p* = 0.041) as well as others (OR 6.96, *p* = 0.010), when compared to MVC. There were no significant associations between a high ISS and males, the type of trauma mechanism including physical violence, stab injury and jumping as well as type of CT examination (whole-body vs. selective CT).

**Table 3:** Multivariable adjusted logistic regression model predicting ISS ≥ 9

	OR (95% CI)	*p*-value
Age	1.02 (1.01; 1.03)	**< 0.001**
Males	1.54 (1.00; 2.37)	0.051
Year		
2009	Ref.	
2013	1.27 (0.71; 2.25)	0.421
2017	0.48 (0.27; 0.83)	**0.009**
Type of incident		
Fall < 3 m	3.84 (1.94; 7.60)	**< 0.001**
Fall > 3 m	6.22 (2.78; 13.9)	**< 0.001**
Motor vehicle collision	Ref.	
Motorbike accident	2.67 (1.05; 6.75)	**0.038**
Bicycle accident	3.65 (1.63; 8.14)	**0.002**
Pedestrian accident	7.46 (1.24; 45.01)	**0.028**
Other traffic accidents	3.14 (1.05; 9.36)	**0.041**
Physical violence	2.23 (0.54; 9.19)	0.268
Stab injury	4.07 (0.59; 28.16)	0.155
Jumping	7.79 (0.61; 99.15)	0.114
Others	6.96 (1.60; 30.27)	**0.010**
Unknown	9.74 (3.53; 26.91)	**< 0.001**
CT examination		
Whole-body CT	1.51 (0.91; 2.49)	0.111
Selective CT	Ref.	

Odds ratios (OR) are from multivariable adjusted logistic regression model

CI: confidence interval

*P*-Values < 0.05 appear bold

## Discussion

Our results demonstrate an increase in CT examinations of acute adult trauma patients at our hospital regarding the year 2009, 2013 and 2017. Simultaneously, we found a significant increase in the mean age of these patients. In contrast, the severity of the traumatic injury of patients receiving WBCT was substantially decreasing over time. We specifically observed a significant decrease of ISS in the younger age groups and women as well as in MVC patients. Further analysis showed that trauma patients with a high ISS were associated with older age and with the type of incident being a fall or unknown at the time of the CT examination.

Multiple studies have reported similar trends in the early 2000s regarding increasing CT utilization at emergency departments [3, 13, 14] and various factors were discussed, which include the increased availability of CT scanners [15], their proximity to EDs, the improved speed of new-generation scanners [16], as well as defensive medicine practice and pressure to decrease patient visit time [13, 17]. In current literature from 2007 and on, the increase in emergency department advanced imaging rates (MRI and CT) appears to be smaller but still continues to rise [18]. Some argue the slower rise in advanced ED imaging after 2007 may be secondary to rising healthcare costs, recognition of overdiagnosis and increased attention to cumulative medical radiation exposure [3, 19]. For example, an observational study by Asha et al. in 2012, calculated that the adjusted odds of receiving a radiation dose of 20 mSv was 2.2 times higher after the introduction of WBCT compared to odds before, with little evidence of reduction in missed injuries [20]. Increasing use of WBCT also results in more patients with incidental findings. Previous studies have reported the detection of incidental findings in 45–53% of trauma patients [21–23]. In addition, a secondary analysis of the REACT-2 multicentre randomized controlled trial found that incidental findings occurred in 43% of the trauma patients with WBCT compared to 33% with selective CT [24]. Incidental findings can provide the advantage of earlier diagnosis of malignancy or vascular disease, however, it should be taken into consideration that they can also result in concerns for the patient, unnecessary follow-up investigations and extra health care costs.

Nevertheless, the increase of trauma CT examinations in recent years similar to our study may also be influenced by the results of the large-scale registry (TraumaRegister DGU®) of the German Trauma society in 2009 and 2013, which suggested a significantly decreased risk-adjusted ratio of observed to expected deaths with primary WBCT (8, 9). In 2016 however, the only prospective randomized trial (REACT-2) about the benefit of WBCT so far reported no reduction in mortality comparing WBCT to a standard work-up with conventional imaging supplemented with selective CT scanning. Interestingly, the median ISS of the patients receiving WBCT compared to standard work-up was 20 compared to 19 [10], whereas the trauma patients with WBCT of the TraumaRegister DGU® had a mean ISS of 32 compared to 28 of patients with non-WBCT [8, 9]. These contradictory results could therefore be due to difference of the study cohort regarding the ISS. Despite the results of REACT-2 [10], we did not see a decrease in WBCT examinations at our hospital the following year. However, trauma patients with WBCT examinations at our hospital had a considerably lower mean ISS (17.1 in 2009) compared to the study cohort of the TraumaRegister DGU® (32 in 2009), even substantially decreasing over time (13.9 in 2013 and 11.7 in 2017). Therefore, the results of the TraumaRegister DGU® suggesting a benefit of primary WBCT for trauma patients might not be applicable to our hospital as well as other medical facilities with a generally lower degree of injury of trauma patients receiving WBCT [8, 9].

In our study we found a significant decrease in ISS especially in the younger age groups, who received immediate trauma CT scans (18 to 24 and 25 to 49 years) but not in the older age groups (50 to 69 years and older). Since the use of CT is increasing worldwide, it is very likely that they will undergo several other CT examinations in their lifetime. Thus, a higher risk of cancer due to radiation exposure has to be taken into consideration for this younger patient group [25]. Furthermore, a significant decrease of ISS was observed in women receiving trauma CT examinations. This is especially surprising since several studies have demonstrated that female gender may have a protective effect regarding traumatic injuries and their resulting in-hospital mortality [26–29] Therefore a gender-specific guideline for CT examinations of trauma patients could be advisable. However more research is needed to examine the physiologic background of this protective effect of female gender in trauma. Interestingly, we also found a significant decrease of ISS in trauma patients, who suffered from MVC and therefore underwent CT examination. Similarly, a time trend analysis of road accidents in Germany regarding the period 1991 until 2011 showed a decrease in injury severity and mortality [30]. Reasons considered for a decrease in motor vehicle-related injury of trauma patients include an increased use of seat belts and airbags [31] as well as improvement in vehicle and road safety [32, 33]. Another reason, however only specific to the region, could be the S3 guideline of the German Trauma Society, published in 2016, which recommended that all patients involved in road traffic accidents with a velocity > 30 km/h to be treated in an emergency room irrespective of their injuries [34]. However, the publications on which this part of the guideline was based date back to the years 1988–2006, whereby their data go back to the 1970s. Further technical developments in vehicle and road safety were therefore not covered by the guideline [35]. The current S3 guideline is however more cautious about the uncritical adoption of the alarm indicators for shock room treatment and they should not automatically be used to determine the indication for a whole-body CT [36]. This is supported by a recent study by Belabbas et al. showing that in patients with normal physical examination of the trunk after a motor vehicle crash, WBCT had no benefit in patients whose severity of trauma was only determined by mechanism of the crash [25].

Similar results to our study, which showed a decrease of ISS in trauma patient and a simultaneous increase of CT examinations, have emanated from the study by Salastekar et al. They found a significant increase of chest and abdominopelvic CT for trauma patients from 2011 to 2018 in the United States. The increase was more pronounced for minor trauma (ISS ≤ 3) than for intermediate (3 < ISS ≤ 15) or major trauma (ISS > 15), with up to a threefold increase for minor injuries [37]. Furthermore, Tong GE et al. found an increase of CT examinations for patients with minor trauma by calculating a 1.97-fold increase in CT from 2005 to 2013, observed in over 8.5 million Californian patients with an ISS smaller than 9. Even after adjusted analysis of independent predictors of increased CT utilization, increasing year was still found to be significantly associated with higher rates of CT examinations [38]. Our data also demonstrated that the mean age of trauma patients increased with time. This result is in line with data from the National Trauma Database in the USA from 2005 to 2015 and the Trauma Register DGU® in Germany from 1994 to 2012 which showed that the mean age of trauma patients and the proportion of geriatric trauma patients are increasing [39]. Using a multivariable adjusted logistic regression model, we identified predicting factors associated with a high ISS in trauma patients, which included older age and the type of incident being a fall. Our results coincide with findings that geriatric trauma patients have a worse prognosis compared to adult trauma patients regarding longer ICU and hospital stays, risk of severe disability and mortality [40–42]. This might also explain the association between a high injury severity and the mechanism of the injury being a fall, since fall is the most common mechanism of injury and the leading cause of trauma-related deaths in geriatric trauma patients [43–45].

Our study has several limitations. First, the study was conducted at a single center and therefore may not be applicable to other settings, especially countries with different trauma guidelines. Additionally, the retrospective design of this study did not allow evaluating the impact of WBCT on trauma patients, regarding further therapeutic management and outcome. We chose the year 2017 as the endpoint of our study since our hospital underwent major conversion work in 2018 and following years with expansion and restructuring of the emergency department, which could bias the results of this study. Furthermore, our study depends on the accuracy of the ISS, which might not completely capture the clinical picture as well as other aspects of a patient’s condition that are not incorporated into this scoring system [38, 46]. In our study mean ISS decreased significantly over time, however, this could be due to various external factors, which we did not control for.

In conclusion, there has been a significant decrease in ISS and a simultaneous increase of WBCT examinations of trauma patients over the years. Therefore, results of previous studies suggesting a benefit of primary WBCT for trauma patients with higher ISS might not be applicable since they might not reflect the current patient population presented at the trauma bay. A decrease in ISS was especially found in trauma patients who were female, of younger age and who suffered from MVC. We therefore recommend further research to focus on a more precise selection of trauma patients benefitting from WBCT to prevent increased radiation dose. Furthermore, older age and fall, as a trauma mechanism, predicted a high ISS in our study. These results further emphasize the importance of prevention of fall or tumble for elderly people.

## Abbreviations

AIS: Abbreviated injury scale
CI: Confidence interval
CT: Computed tomography
ICU: Intensive care unit
ISS: Injury severity score
OR: Odds ratios
SCT: Selective computed tomography
WBCT: Whole-body computed tomography
